# Clinical Evaluation of a Mobile Sensor-Based Gait Analysis Method for Outcome Measurement after Knee Arthroplasty

**DOI:** 10.3390/s140915953

**Published:** 2014-08-28

**Authors:** Tilman Calliess, Raphael Bocklage, Roman Karkosch, Michael Marschollek, Henning Windhagen, Mareike Schulze

**Affiliations:** 1 Department for Orthopaedic Surgery at the Annastift, Hannover Medical School, 30625 Hannover, Germany; E-Mails: raphael.bocklage@gmail.com (R.B.); roman_karkosch@web.de (R.K.); henning.windhagen@ddh-gruppe.de (H.W.); 2 Hannover Medical School, Peter L. Reichertz Institute for Medical Informatics, University of Braunschweig-Institute of Technology and Hannover Medical School, 30625 Hannover, Germany; E-Mails: michael.marschollek@plri.de (M.M.); mareike.schulze@plri.de (M.S.)

**Keywords:** sensor-based mobile gait analysis, outcome measurement, total knee arthroplasty, sports ability

## Abstract

Clinical scores and motion-capturing gait analysis are today's gold standard for outcome measurement after knee arthroplasty, although they are criticized for bias and their ability to reflect patients' actual quality of life has been questioned. In this context, mobile gait analysis systems have been introduced to overcome some of these limitations. This study used a previously developed mobile gait analysis system comprising three inertial sensor units to evaluate daily activities and sports. The sensors were taped to the lumbosacral junction and the thigh and shank of the affected limb. The annotated raw data was evaluated using our validated proprietary software. Six patients undergoing knee arthroplasty were examined the day before and 12 months after surgery. All patients reported a satisfactory outcome, although four patients still had limitations in their desired activities. In this context, feasible running speed demonstrated a good correlation with reported impairments in sports-related activities. Notably, knee flexion angle while descending stairs and the ability to stop abruptly when running exhibited good correlation with the clinical stability and proprioception of the knee. Moreover, fatigue effects were displayed in some patients. The introduced system appears to be suitable for outcome measurement after knee arthroplasty and has the potential to overcome some of the limitations of stationary gait labs while gathering additional meaningful parameters regarding the force limits of the knee.

## 1. Introduction

Self-questionnaires, clinical function scores, and motion-capturing gait analysis are today's gold standard for outcome measurement after total knee arthroplasty (TKA). However, critics question the possible bias of these techniques, as well as whether they reflect truly relevant issues regarding patients' quality of life [1–4].

As the overall incidence of TKA increases, young and active patients in particular represent a growing subpopulation. These patients present a special task for orthopaedic surgeons and for outcome analysis, because of their high demands regarding return to work or even sports.

While self-questionnaires are mainly intended to reflect patients' satisfaction, pain, or impairments in activities of daily living (ADL), clinical scores are designed to provide a picture of the functional outcome. Interestingly, there is often no correlation between objective joint function and patient satisfaction [5]. This is especially true regarding the referred young and active patients, such that some authors question the meaningfulness of these methods for said cohort [1]. For example, a patient might have excellent objective function, but still be unable to perform desired sports-related activities. This discrepancy could be because many of the characteristics collected are static parameters, such as range of motion, stability, or limb alignment, and often provide no information about dynamic behaviour.

In this context, gait analysis as a dynamic examination may provide an objective functional outcome measurement that has a better correlation with the subjective impairments experienced in daily life. Several studies demonstrate good correlation between gait parameters, patient mobility, and satisfaction [6]. However, the common stationary gait lab implements an artificial, observed environment that only allows the simulation of specified test setups, which again may not provide an adequate picture of ADL or sports ability. For example, because most gait analyses examine walking distances of less than 15 m, several effects (including fatigue) cannot be studied appropriately. Even stair climbing or said sportive activities cannot be simulated adequately.

Some unobtrusive sensor-based mobile systems have been introduced in the past as an alternative to the stationary gait lab analysis [4,7]. Although these systems are by definition not as sophisticated as stationary gait measurements, they are able to display the common spatiotemporal parameters of gait (e.g., speed, cadence, stride length) with sufficient accuracy [8–11]. Moreover, they are able to display activity and gait during daily living [12,13]. Given these characteristics, such systems are of great interest for further outcome measurement after TKA. However, the currently available systems have mainly been introduced to monitor general rehabilitation [14], geriatric patients [15], or neurological patients [16], and none of them is focused on displaying relevant function parameters of the knee. Therefore, our group has developed and validated a specific sensor-based mobile gait analysis system [17] that is designed to assess specific knee outcome parameters. We recently conducted a validation study of our system with video cameras and a Vicon motion-capturing gait lab [18,19].

The aim of this study was to evaluate our system regarding the outcome measurement of TKA in a clinical setting. Our focus was on the system's ability to display specific knee parameters and gait information during ADLs and sportive activities. The ability of parameters to assess sports ability should be identified according to their correlation with the clinical outcome data. Our hypothesis was that activities with load limits for the knee joint (e.g., running or abrupt stops) would correlate more strongly with each patient's sports ability than the evaluation of normal gait alone.

## 2. Experimental Methodology

Six patients (three men and three women) with primary osteoarthritis of the knee undergoing knee arthroplasty in our department were enrolled in this study. The inclusion criteria were defined as body mass index <30 and intact cruciate and collateral ligaments. In addition, we were focusing on young and active patients between 50 and 70 years of age with a University of California, Los Angeles (UCLA) activity score > 5 prior to surgery. Four patients received total bi-condylar knee replacement (TKA) (Stryker Triathlon^®^, Kalamazoo, MI, USA), and two patients underwent medial uni-compartmental knee replacement (UKA) (Stryker Triathlon PKR^®^, Kalamazoo, MI, USA) according to the individual indication for surgery.

**Figure 1.**
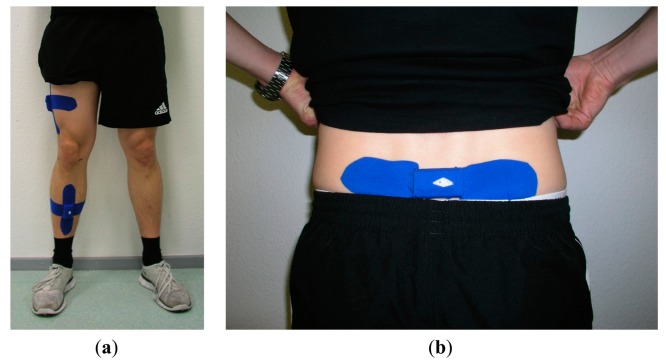
Elastic therapeutic tape was used to attach all sensors. (**a**) Display of the positions of the thigh and shank sensors; (**b**) Display of the pelvic sensor position.

The day before surgery and 12 months postoperatively, clinical orthopaedic examination of the knee joint with acquisition of the standard Knee Society Score (KSS, max. 100 Points) and Oxford Knee Score (OKS, max. 48 points) were conducted. In addition, gait analysis was performed using a self-developed and previously validated mobile system, as described previously [18]. In brief, the system consisted of three commercially available sensor units (SHIMMER 2R, Shimmer/Realtime Technologies, Dublin, Ireland), each equipped with a triaxial accelerometer, a triaxial gyroscope, and a triaxial magnetometer (9 DoF). These units were attached to the patient using elastic therapeutic tape (Temtex^®^, Guri City, Korea). The first sensor was placed dorsally on the lumbosacral junction. The second sensor was placed on the lateral thigh, on the iliotibial band 20 cm cranial of the knee joint gap. The third sensor was situated at the medial aspect of the sink on the tibia 20 cm caudal of the knee joint gap (Figure 1). This placement was to ensure that the sensors were positioned identically for different test scenarios and to minimize motion relative to the skeleton underneath.

After the previously calibrated sensors were positioned as described, raw data was streamed via Bluetooth^®^ to a tablet PC in real time. Data were recorded and synchronised using our self-developed software (developed in the platform-independent programming language Java using the RXTX interface standard for the Bluetooth^®^ connection; software and hardware architecture are described in [18]). First, the initialized sensor positions were calibrated in three different knee flexion angles in supine position and while standing upright for 5 s. The standard test protocol began with a 2-min warm-up walk to adjust the sensors and setting. Next, several exercises were performed: (1) the “time up and go” (TUG) test with three repetitions; (2) a 100-m walk on an even surface at a self-selected speed; (3) a 50-m run, as fast as possible; (4) a maximum acceleration sprint with an abrupt stop; and (5) four flights of stairs (up and down). Data was collected during each test and during the transfer periods. In total, the overall test period was approximately 30 min of continuous recording per patient and session. The system itself is capable of approximately 4 h of continuous data acquisition in this test setup. To facilitate automatic data evaluation, recorded data was annotated online in our software by a technician.

Our validated proprietary software, which was developed in the platform-independent programming language R for statistical computing and graphics, was used to determine general parameters of gait (e.g., walking speed, running speed, step cadence, step length and step symmetry). A detailed description of self-developed algorithms from our institute can be found in [20,21]. To achieve the maximum possible precision, we computed speed, cadence, and step length based on a predetermined path length that was used in accordance with the study protocol and annotated while recording. Additionally, the maximum knee flexion angles (mean and range) during walking, stair climbing, and position transfer (from sitting to standing) were calculated from the relative positions of the thigh and shank sensors (as described in more detail in [17–19]). To ensure that the results are representative, initial and final strides were excluded from the analysis. Moreover, the angular velocity in the knee was outputted as a parameter for the power of acceleration and deceleration. The pre-processed data graphs were subjected to additional mathematic analysis to display fatigue effects and to identify evasive movements caused by instability in the knee joint.

## 3. Results and Discussion

The patients' demographics and clinical outcome parameters of the six enrolled patients are displayed in Table 1. Five of the six patients exhibited significant improvement in knee function, as demonstrated by the KSS and OKS scores before and after surgery. The one patient that did not benefit from the operation experienced ongoing pain and had clinically relevant anteroposterior instability in the knee. Interestingly, all patients reported being satisfied (*n* = 3, including Patient 1), or very satisfied (*n* = 3) with their knee replacement 12 months postoperatively. When patients were asked whether they reached the expected level of activity at 12 months after surgery, three patients answered positively and three answered negatively. However, four patients reported persistent limitations in their desired activities, while two patients (Patient 2 and Patient 3) felt no restrictions in their activity level.

For all patients, gait analysis indicated post-surgical improvements in walking speed (mean +0.225 m/s), cadence (mean +10 steps/min) and step length (mean +0.1 m) (Table 2). However, the actual values and the individual effect differed in every patient. The patient with the poorest outcome regarding the clinical scores was also the patient with lowest outcome regarding the gait parameters (walking speed, stride length, running speed); however, interestingly his improvements from pre- to postop in gait analysis were equal to those of other subjects. Overall, the improvement in the rating of the outcome scores (Table 1) was not necessarily reflected in the normal gait parameters (Table 2). For example, Patient 3 experienced no difference in walking speed, cadence, or stride length at 12 postoperative months, although his KSS and OKS scores improved from poor/fair to excellent. This was only reflected in his improvement in maximum running speed. Same applies for Patient 2 with a high correlation between the improvement in the OKS score and running speed. The other patients showed also a high correlation between the actual running speed and the OKS score.

The maximum running speed had also a good correlation to the self-reported patient satisfaction and activity level. Patients 1, 4 and 6 reported to be satisfied, but not very satisfied, and to be less active than expected. In contrast to this, the patients with the best improvement in the running ability were very satisfied with their personal outcome after TKA. In the running test, two patients reached approximately 10 km/h (=2.78 m/s) at 12 postoperative months (Patients 2 and 3), which was almost double their normal walking speed. These patients were the ones that reported no restrictions regarding their activity level and sports ability.

The maximum knee flexion angle during walking did not differ greatly in any of the patients before *vs.* after surgery. The mean flexion was 57.8° (range 44°–71°) in the arthritic condition and 61.2° (range 44°–81°) after knee replacement. Again, the inter-personal differences were quite large and there were no visible correlation between the outcome scores and these parameters.

In contrast to the demonstrated progress in walking and running, the ability to ascend/descend stairs was only very slightly improved after knee replacement compared with the operative condition with respect to speed (Table 3). Patients needed an average of 0.05 s less per step to ascend and 0.07 s less per step to descend stairs. As in the task of walking on a level surface, there was no significant improvement of knee flexion while ascending (on average, 77° to 79°) or descending (on average, 72° to 74°) stairs. However, we observed improvement in the initial knee flexion at heel strike for every patient (Table 3).

In the instrumented TUG test, the time for transition from sitting to standing was reduced from 2.2 s before surgery to 1.7 s at 12 postoperative months; the transition from standing to sitting was reduced from 2.5 s before surgery to 1.9 s at 12 postoperative months. Moreover, we detected less evasive movements during the transfer post-surgery (leaning forward, bending sidewise), and they were less than 10° on average. The maximum knee flexion during transfer improved by an average of approximately 25° (range 4°–58°).

The maximum power of acceleration exhibited in the start-to-run test is displayed in Table 4. Power of acceleration deteriorated in most patients. In contrast, the maximum power of deceleration (which indicates the ability to execute an abrupt stop or turn) improved after knee replacement in every case except for Patient 1, who had pre-existing anteroposterior instability. This parameter again showed a high correlation to the clinical outcome scores with the highest improvement for Patients 2, 3 and 5, reaching the highest OKS scores and showing the best improvements in running speed.

Additional mathematic analysis of the pre-processed data revealed fatigue effects in several patients. We used the pelvic gyroscope data to evaluate increasing evasive movements during the task of walking on a level surface. We also employed a comparison analysis of the amplitudes of the gyroscope data from the shank sensor as a surrogate parameter to evaluate the stability of the knee joint.

**Figure 2.**
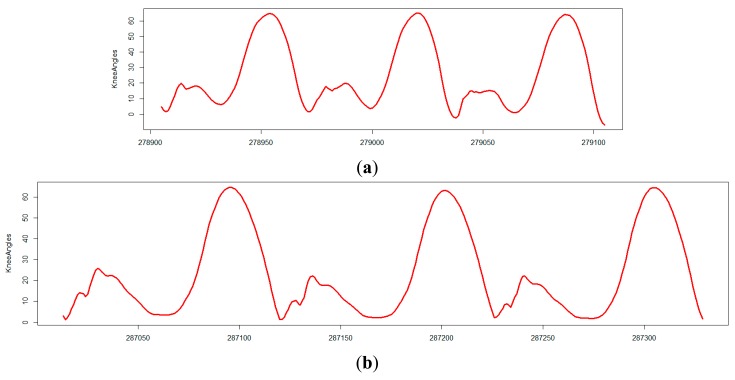
The knee angle curves of Patient 3 during the task of walking on a level surface. The arthritic instability of the knee exhibited by Patient 3 before surgery (**a**; standing leg remains almost stiff) was barely visible after surgery (**b**; normal heel-toe stride in stance phase).

For example, Patient 3 exhibited an amplitude peak of the gyroscope of 563 in the coronal plane vector during the single leg support phase in the shank, which was reduced to 302 after surgery. This information is meant as an indication of relevant mediolateral instability of the knee joint, which was no longer visible after surgery. This change can also be observed in the comparison of knee angle curves while walking before and after surgery (Figure 2; see [22]). As reported, Patient 1 had clinically apparent anteroposterior instability after surgery; again, this was apparent in the comparison of the amplitude of the gyroscope in the sagittal plane before and after surgery. This finding correlated with the only negative trend in the power of deceleration.

Our introduced sensor-based mobile gait analysis system appears to be a suitable tool for outcome measurement after knee arthroplasty. In this study, we were able to demonstrate that the system is appropriate for a clinical application in which we performed gait analyses before and after knee surgery in active patients. We were able to examine several typical ADLs, as well as sportive activities. The system is able to evaluate the standard parameters of gait, including walking speed, cadence, stride length, and step symmetry, as previously reported. We were also able to show that activities with load limits for the knee joint, such as walking downstairs, running, and stopping abruptly, can provide additional information about knee function and the patient's confidence in the knee replacement. There was a strong correlation between these parameters and self-reporting function scores (OKS), especially regarding patients' sports ability. This correlation was not observed in the normal walking situation (*i.e.*, while walking at a self-selected, comfortable speed).

In comparison to the gold standard of gait analysis—the stationary marker-based motion capturing gait lab—these parameters of interest are not part of the standard test protocol, and are difficult to display in such a setting. Therefore, we believe that our system has the potential to overcome some of the limitations of stationary gait measurements in outcome analysis after surgery, particularly with regard to physically demanding and active patients. Fatigue effects could also be more appropriately displayed and followed over time in our setting than in stationary gait labs.

Today, several mobile gait systems are available on the market or at least described in research papers [8,11,16,23–25]. Some of the systems that are currently available on the market use only one pelvic sensor to display the spatiotemporal gait or activity parameters, and no information about knee function can be obtained with that setup [8,11]. These systems are of limited use for the outcome analysis of different treatment strategies or different prosthetic designs, as relevant data about joint velocity or stability cannot be evaluated. The more sophisticated systems, which have two, three, or more sensors placed on the pelvis and the extremities, are theoretically able to monitor knee range of motion and said parameters [16,23,25]. However, the accuracy of the data must be questioned. First, to our knowledge only two systems have separate thigh and shank sensors and the ability to determine the real knee range of motion between the two units [23,25]. All of the other systems evaluate only the shank range of motion and use it to calculate the range of motion of the knee joint. Alternatively, they use an electronic goniometer that does not fulfil the definition of an unobtrusive device [9]. Second, the sensors of most other systems are secured to the body with straps [16,25]. No specific position is defined, and a broad range of relative motion between the sensor and the body is possible, which would certainly affect the accuracy of the data. Our system addresses this problem by using elastic therapeutic tape to secure the sensors, which guarantees a stable, defined sensor position. In comparison with competing commercially available systems, ours is the only one that focuses on special knee parameters (e.g., knee flexion profile during activities, velocity during activities, and knee stability). We were able to demonstrate that these parameters might be of special interest regarding the outcome analysis of knee prosthesis for active patients because of their high correlation with sports ability.

In the current literature, gait speed is often referred to as one of the primary outcome parameters after total joint replacement [26]. However, some authors point out that gait speed also depends on the distance that must be traversed [4]. Therefore, this parameter might be affected more in the gait lab than in our setting. Furthermore, we were able to demonstrate that walking on a level surface at normal speed correlates less strongly with clinical outcome scores, patient satisfaction, and particularly sports ability than does the maximum speed during running. Hence, we consider this parameter meaningful regarding the outcome measurement, especially for very active and physically demanding patients. Interestingly, all of the study participants exhibited a postoperative reduction of maximum acceleration in the knee joint in the start-to-run test. This result might be attributed in part to the lower proprioception of the knee replacement compared with the native joint. In contrast, the power of deceleration increased in all patients, with the exception of one patient with postoperative knee instability. We believe that power of deceleration could act as a surrogate parameter for knee joint stability after joint replacement.

The identified changes in knee flexion before *vs.* after surgery are consistent with the literature, and in some cases slightly better than published reports [27]. This result may be attributed to the relative youth and relatively high activity level of our study cohort. The most interesting effect we observed was an obvious increase in knee flexion at heel strike while descending the stairs. From a clinical standpoint, we see this as a critical parameter for displaying load-ache and the stability of the knee joint during flexion. Patient 1, with postoperative pain and anteroposterior instability, exhibited the least improvement in this parameter.

Regarding the other parameters mentioned, our data indicates post-replacement improvements in gait similar to the current literature [27]. As in other studies, the inter-individual range of the parameters is quite broad, so that no significant effects or correlations with satisfied or unsatisfied patients have been noted in our small cohort, thus far [28]. This leads to the limitations of our study. First, the present study reports only about the initial experiences and the evaluation of the suitability of our system for clinical outcome measurement after TKA. Only a small cohort of six patients was enrolled in this study. Moreover, we must clarify that our patient selection, which includes only young and active patients, does not represent the common population enrolled for TKA. Therefore, the outlined effects must be validated in a larger patient population in the future. It is likely that some of the tests or findings will not transfer directly to other (especially older or obese) patients. This is particularly true of the newly discussed parameters, such as the power of deceleration in the knee joint and the suggested stability parameters.

Second, in our setup sensors are placed on the affected leg only, and are absent from the unaffected leg. Therefore, no data can be obtained for comparison to the non-operated knee. Placement of sensors on the affected leg alone keeps the system easy to use and allows us to overcome problems with the limited Bluetooth^®^ transmission protocol. Although the parameters of step count [20] and step symmetry [21] can be obtained from the pelvic sensor, no accurate information can be obtained about the single-leg stance of the non-operated side or the double-leg stance. These parameters are often referred to in the literature; however, we do believe that our outputted gait symmetry is an equivalent model with which to evaluate limping. When additional measurements of standardized gait are available for each patient, it would also be possible to precisely estimate mean step length for straight-line walking (which is based on an individual adjusted inverted pendulum model) from the pelvic sensor alone [29].

## 4. Conclusions/Outlook

In summary, our system appears to be a suitable tool for outcome measurement after knee arthroplasty and has the potential to overcome some of the limitations of stationary gait measurements. We were able to demonstrate that activities that include load limits for the knee joint, such as stopping abruptly or running, correlate more strongly and meaningfully to the functional outcome and sports ability of the active patient than the evaluation of normal gait alone. Therefore, our primary hypothesis was fulfilled. However, more relevant data must be obtained for additional validation of the described parameters and effects. Additional clinical studies analysing patients undergoing knee replacement are needed.

## Figures and Tables

**Table 1.** Patients' demographics and clinical outcome parameters/scores before (Pre) and 12 months after (Post) surgery.

Pat. ID	Side	Knee System	Age	Sex	BMI	UCLA Score	Pre-KSS	Post-KSS	Pre-OKS	Post-OKS
1	R	UKA	52	M	29.4	7	38	37	18	22
							(poor)	(poor)	(poor)	(fair)

2	R	TKA	57	F	24.2	7	23	91	29	44
							(poor)	(excellent)	(fair)	(excellent)

3	R	TKA	63	M	22.7	8	33	88	28	43
							(poor)	(excellent)	(fair)	(excellent)

4	L	TKA	68	M	27.1	6	34	80	22	35
							(poor)	(excellent)	(fair)	(good)

5	L	TKA	57	F	28.9	7	44	79	34	38
							(poor)	(good)	(good)	(good)

6	L	UKA	64	F	24.5	7	49	82	30	36
							(poor)	(excellent)	(good)	(good)

**Table 2.** Spatiotemporal gait parameters for normal walking and running on a level surface before (Pre) and 12 months after (Post) surgery (mean values).

Pat. ID	Walking Speed			Cadence			Step Length			Running Speed		
			
	(m/s)			(steps/min)			(m)			(m/s)		
			
	Pre	Post	Δ	Pre	Post	Δ	Pre	Post	Λ	Pre	Post	Δ
1	0.92	1.25	0.33	104	122	18	0.5	0.6	0.1	1.06	1.36	0.3
2	1.44	1.67	0.23	117	126	9	0.7	0.8	0.1	2.08	2.69	0.61
3	1.5	1.5	0	114	116	2	0.8	0.8	0	2.42	3.03	0.61
4	1	1.44	0.44	100	124	24	0.6	0.7	0.1	1.58	1.75	0.17
5	1.22	1.39	0.17	116	120	4	0.6	0.7	0.1	1.25	2.11	0.86
6	1.25	1.39	0.14	111	116	5	0.7	0.7	0	1.58	1.86	0.28

**Table 3.** Mean values of the evaluated gait parameters during stair climbing before (Pre) and after (Post) surgery.

Pat. ID	Time per Stair Step			Time per Stair Step			Max. Knee Flexion			Max. Knee Flexion			Knee Flexion at Heelstrike		
				
	Ascending (s)			Descending (s)			Ascending [°]			Descending (°)			Descending (°)		
				
	Pre	Post	Δ	Pre	Post	Δ	Pre	Post	Δ	Pre	Post	Δ	Pre	Post	Δ
1	0.6	0.58	−0.02	0.54	0.51	−0.03	78	84	6	81	84	3	25	27	2
2	0.5	0.43	−0.07	0.51	0.41	−0.1	74	77	3	72	69	−3	22	26	4
3	0.51	0.5	−0.01	0.49	0.4	−0.09	83	78	−5	70	69	−1	6	20	14
4	0.75	0.64	−0.11	0.59	0.58	−0.01	49	87	38	36	74	38	8	12	4
5	0.57	0.5	−0.07	0.53	0.44	−0.09	93	75	−18	101	78	−23	18	24	6
6	0.66	0.67	0.01	0.65	0.53	−0.12	86	72	−14	84	84	0	7	25	18

**Table 4.** Knee velocity parameters during maximum acceleration and abrupt cessation of running before (Pre) and after (Post) surgery.

Patient ID	Power of Acceleration			Power of Deceleration		
	
	(Vector)			(Vector)		
	
	Pre	Post	Δ	Pre	Post	Δ
1	2	1.4	−0.6	4.2	3.1	−1.1
2	5.7	2.6	−3.1	2.1	3.4	1.3
3	7.4	4.2	−3.2	1.7	2.9	1.2
4	3.4	3.1	−0.3	1.9	2.3	0.4
5	2.2	3.3	1.1	1.8	3.4	1.6
6	1.8	2.8	1	1.9	2.5	0.6
